# A Substitution in the Ligand Binding Domain of the Porcine Glucocorticoid Receptor Affects Activity of the Adrenal Gland

**DOI:** 10.1371/journal.pone.0045518

**Published:** 2012-09-18

**Authors:** Eduard Murani, Henry Reyer, Siriluck Ponsuksili, Stephan Fritschka, Klaus Wimmers

**Affiliations:** 1 Research Unit Molecular Biology, Leibniz Institute for Farm Animal Biology (FBN), Dummerstorf, Germany; 2 Research Group Functional Genome Analysis, Leibniz Institute for Farm Animal Biology (FBN), Dummerstorf, Germany; Morehouse School of Medicine, United States of America

## Abstract

Glucocorticoids produced in the adrenal cortex under the control of the hypothalamic-pituitary axis play a vital role in the maintenance of basal and stress-related homeostasis and influence health and well-being. To identify loci affecting regulation of the hypothalamic-pituitary-adrenal (HPA) axis in the pig we performed a genome-wide association study for two parameters of acute and long-term adrenal activity: plasma cortisol level and adrenal weight. We detected a major quantitative trait locus at the position of the glucocorticoid receptor gene (*NR3C1*) – a key regulator of HPA axis activity. To determine the causal variant(s), we resequenced the coding region of *NR3C1* and found three missense single nucleotide polymorphisms (SNPs). SNP c.1829C>T, leading to a p.Ala610Val substitution in the ligand binding domain, showed large (about 0.6× and 1.2× phenotypic standard deviations for cortisol level and adrenal weight, respectively), and highly significant (2.1E-39≤log10(1/p)≤1.7E+0) negative effects on both traits. We were able to replicate the association in three commercial pig populations with different breed origins. We analyzed effects of the p.Ala610Val substitution on glucocorticoid-induced transcriptional activity of porcine glucocorticoid receptor (GR) *in vitro* and determined that the substitution introduced by SNP c.1829C>T increased sensitivity of GR by about two-fold. Finally, we found that non-coding polymorphisms in linkage disequilibrium with SNP c.1829C>T have only a minor effect on the expression of *NR3C1* in tissues related to the HPA axis. Our findings provide compelling evidence that SNP c.1829C>T in porcine *NR3C1* is a gain-of-function mutation with a major effect on the activity of the adrenal gland. Pigs carrying this SNP could provide a new animal model to study neurobiological and physiological consequences of genetically based GR hypersensitivity and adrenal hypofunction.

## Introduction

Hormones produced by the adrenal gland (corticosteroids and catecholamines) play a vital role in maintaining homeostasis, particularly during stress. Glucocorticoids (in the pig, cortisol) produced by adrenocortical cells of the *zona fasciculata* under the control of the hypothalamic-pituitary axis facilitate coping with stress and adaptation by influencing various neurobiological, metabolic, and immune processes [1]. Dysfunction of the hypothalamic-pituitary-adrenal (HPA) axis therefore has adverse effects on health status and well-being. In humans, for example, dysregulation of glucocorticoid secretion and signaling has been implicated in the pathogenesis of mood disorders and metabolic syndrome [2], [3].

In different species, including the pig, glucocorticoid secretion shows large inter-individual variation that has a considerable genetic component [4], [5]. Little is known about the underlying genetic variants; however, their identification and utilization as simple DNA markers in molecular breeding is a promising approach to improving adaptation potential, health, and welfare in pigs and in farm animals in general [6]. Indeed, implementation of traditional breeding programs for these traits, based on phenotypic data, is hampered by their difficult and expensive ascertainment [7].

To identify genes affecting acute and long-term regulation of HPA axis activity in the pig, we previously analyzed polymorphisms in ten functional candidates for potential associations with plasma cortisol level and adrenal weight (herein summarily designated as adrenal activity) in commercial crossbreds. The glucocorticoid receptor gene (*nuclear receptor subfamily 3, group C, member 1*; *NR3C1*) was identified as a promising candidate because we found significant association of a non-coding variant (SNP c.*2122G>A) with both traits analyzed [8]. Glucocorticoid receptor (GR) is a ligand-activated transcription factor transducing glucocorticoid signals in almost all organs and tissues, including hypothalamus and pituitary where it mediates feedback inhibition of HPA axis activity by downregulating expression and release of corticotropin-releasing hormone (CRH) and corticotropin (ACTH), respectively [9]. In humans several rare mutations and common polymorphisms of *NR3C1*, mainly non-synonymous, have been shown to generate diversity in GR signaling and to contribute to inter-individual variation in glucocorticoid secretion [10], [11].

To facilitate a more comprehensive understanding of the genetic architecture of HPA axis activity, here we performed a genome-wide association study (GWAS). This uncovered *NR3C1* as a major QTL for plasma cortisol level and adrenal weight.

To identify the causal variant(s) we first concentrated on the analysis of non-synonymous polymorphisms of *NR3C1* because they appear to be the main source of inter-individual variation in GR function, and because they are most amenable to functional interpretation. We present here strong genetic and functional evidence that a non-synonymous SNP (c.1829C>T) in *NR3C1*, leading to the gain-of-function substitution p.Ala610Val in the ligand binding domain of GR, is indeed causally involved.

## Results

### A genome-wide association study identifies a major quantitative trait locus for adrenal activity at the *NR3C1* position

The GWAS was performed in a population of commercial German Landrace pigs (n = 564), which were genotyped using the PorcineSNP60 BeadChip (Illumina) and phenotyped with respect to plasma cortisol level and adrenal weight (overview of all phenotypic data used in this study is presented in Table S1). After a filtering step, genotype information for 42,359 SNPs that mapped to the 18 autosomes (Sscrofa 10.2) was used.

**Figure 1:**
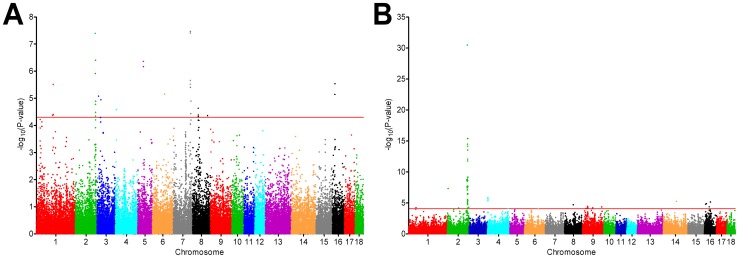
Manhattan plot of the genome-wide association analysis of plasma cortisol levels (A) and adrenal weight (B). The red line indicates the significance threshold corresponding to a genome-wide false discovery rate q-value ≤0.05.

A total of 37 SNPs mapping to nine chromosomes were significantly associated with plasma cortisol level at the genome-wide false discovery rate (FDR) q≤0.05 level (Figure 1A and Table S2). The strongest association signals were found on chromosomes 2 and 7. On chromosome 2 nine significant SNPs, located in an ∼2.6 Mb region between SNPs ALGA0106239 and ALGA0123033, were identified. The two most significantly associated (p = 4.0E-08) SNPs, ALGA0106239 and DRGA0017574, showed complete linkage disequilibrium, and each explained ∼10% of the phenotypic variance (Table S2). On chromosome 7 seven significant SNPs, located in an ∼4.9 Mb region between SNPs H3GA0023063 and MARC0044680, were found. The most significantly associated SNP (p = 3.4E-08), ASGA0036275, explained ∼10% of the phenotypic variance (Table S2).

The GWAS for adrenal weight yielded 62 genome-wide significant SNPs (q≤0.05), located on seven chromosomes (Figure 1B and Table S3). The strongest association signal was found on chromosome 2, where 41 significant SNPs clustered in an ∼9.7 Mb region between SNPs M1GA0024750 and ALGA0123873. However, the majority of the SNPs (35), including all nine SNPs significantly associated with plasma cortisol level, fell in a considerably smaller region (∼3 Mb) between SNPs ALGA0106386 and ALGA0120126. Except on chromosome 2, there was no overlap between the association signals for plasma cortisol level and adrenal weight. SNPs ALGA0106239 and DRGA0017574 again showed the strongest association (p = 3.5E-31), and each explained ∼30% of the phenotypic variance in adrenal weight. Significant SNPs in other genomic regions explained less than 7% of the phenotypic variance (Table S3).

According to the current annotation of the porcine genome (Pre Ensembl Sscrofa 10.2) the ∼3 Mb region flanked by SNPs ALGA0106386 and ALGA0120126 contains eight annotated genes, including *NR3C1*. The two most significant SNPs, ALGA0106239 and DRGA0017574, are located within porcine *NR3C1,* in introns 6 and 5, respectively.

The GWAS thus confirmed association of *NR3C1* with plasma cortisol level and adrenal weight in an additional, purebred, population and suggested that polymorphisms of *NR3C1* account for the largest proportion of the variation in adrenal activity.

### Resequencing of the region encoding the porcine glucocorticoid receptor alpha isoform reveals a potentially functional missense variant

**Figure 2:**
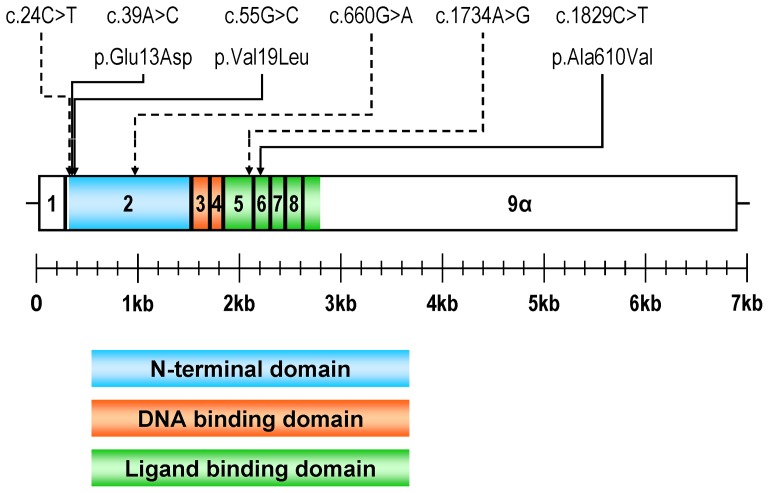
Position of the detected single nucleotide polymorphisms in porcine *NR3C1.* Exons are represented by numbered boxes. Coding region is highlighted by coloring according to the encoded functional domain. Single nucleotide polymorphisms (SNP) and resulting amino acid substitutions are designated according to human genome variation society nomenclature. Synonymous and non-synonymous SNPs are represented by dashed and solid arrows, respectively.

We resequenced the whole ∼2.38 kb region of *NR3C1* encoding the predominant isoform of GR–alpha–in a panel of samples from four different breeds [two each, German Landrace (LR), German Large White (LW), Pietrain (Pi), and Duroc (Du)] and in pooled samples of crossbred animals [Pietrain × (German Large White × German Landrace), noted as PiF1] with extreme phenotypes for cortisol secretion and adrenal weight, respectively (three each for high and low cortisol and heavy or light adrenal gland). We identified a total of six SNPs (Figure 2): three synonymous (c.24C>T, c.660G>A, c.1734A>G) and three missense polymorphisms (c.39A>C, c.55G>C, c.1829C>T).

The missense SNP c.39A>C and c.55G>C lead to amino acid substitutions (p.Glu13Asp and p.Val19Leu, respectively) in the N-terminal domain in a region of GR-alpha showing a relatively low level of amino acid sequence conservation (Figure S1) [12]. The third missense SNP, c.1829C>T, leads to an alanine-to-valine substitution (p.Ala610Val) in helix 5 of the ligand binding domain (GR-LBD) (Figure S1). Crystallographic and mutagenesis studies of human and murine GR-LBD reveal that helix 5 is involved in the formation of the ligand binding pocket of GR-alpha [13] and suggest that p.Ala610Val substitution might enhance affinity and, consequently, responsiveness of GR to glucocorticoids by intensifying van der Waals contacts with the ligand [14].

### SNP c.1829C>T shows consistent association with cortisol level and adrenal weight across different pig breeds

We sought to assess frequency of the missense SNPs in the most important commercial breeds and in populations included in association analyses, specifically in the LR population initially used for the GWAS and in LW and PiF1, which were also phenotyped with respect to plasma cortisol level and adrenal weight. We therefore genotyped 21 to 35 unrelated LR, LW, Pi, and Du pigs. While SNP c.1829C>T segregated in all four breeds, SNP c.55G>C segregated only in Pi. Moreover, in contrast to LR, LW, and Du, the Pi breed was fixed at SNP c.39A>C. With the exceptions of c.39A>C in Du and c.55G>C in Pi, all three missense SNPs segregated at relatively low frequency, generally about 10% or less. Genotyping of additional 92 LR and 45 LW pigs (in total 127 and 69 individuals, or 15% and 25% of the two populations, respectively) again revealed no carriers of SNP c.55G>C, therefore SNP c.55G>C was assumed to be specific for the Pietrain breed. Consequently, SNP c.39A>C and c.1829C>T were genotyped in all three populations (total n = 834 for LR, n = 274 for LW, and n = 537 for PiF1) used for association analyses; SNP c.55G>C was genotyped only in PiF1. In LR and LW the genotype of SNP c.55G>C was assumed to be homozygous GG. Allele distribution of the missense SNPs in the different populations is summarized in Table S4. None of the populations of purebred pigs showed significant deviation from Hardy-Weinberg equilibrium (HWE; p>0.05), indicating that the missense SNPs had no negative impact on viability of their carriers. In PiF1 SNP c.55G>C and c.1829C>T showed significant deviation from HWE (p<0.05), which is most likely a result of crossbreeding because we frequently observe deviation from HWE in this cross (see [8]).

Using the genotypic information collected in LR, LW, and PiF1 we performed population-based single-marker association analysis. Whereas SNP c.39A>C and c.55G>C showed no or only inconsistent associations, SNP c.1829C>T was consistently and, in general, highly significantly associated with decreased cortisol level and adrenal weight in all three populations (Tables 1,2). The estimated allele substitution effect was—with an average of about -0.6 phenotypic standard deviations for cortisol level and about -1.2 phenotypic standard deviations for adrenal weight, respectively—very pronounced. Heterozygous carriers generally showed intermediate values, indicating that SNP c.1829C>T is associated with an additive effect. The estimated phenotypic variance explained by SNP c.1829C>T was ∼10% for cortisol level and ∼25% for adrenal weight, in a similar range as found for the QTL detected by the GWAS.

**Table 1:** Association of the three identified missense SNPs in porcine NR3C1 with plasma cortisol level (ng/ml) in three different commercial populations.

SNP	Population^1^ (n)	p-value	LSM ± SE^2^ (n)			a^3^ (% var^4^)
**c.39A>C**			AA	AC	CC	
	LR (786)	0.906	74.2±1.8 (765)	73.4±7.0 (21)		
	PiF1 (472)	0.048	93.7^a^±1.8 (444)	81.5^b^±6.0 (28)		−0.38 (1.3)
	LW (233)	0.347	81.4±3.6 (218)	89.8±9.0 (15)		
**c.55G>C**			GG	GC	CC	
	PiF1 (472)	0.339	91.8±2.1 (291)	94.8±2.6 (181)		
**c.1829C>T**			CC	CT	TT	
	LR (786)	4.2E-14	78.1^A^±1.8 (661)	57.4^B^±2.9 (121)	31.9^AB^±13.8 (4)	−0.69 (10.5)
	PiF1 (472)	1.0E-06	95.5^A^±1.8 (420)	74.2^B^±4.7 (45)	55.7^B^±11.4 (7)	−0.65 (8.8)
	LW (233)	0.018	85.3^a^±3.7 (186)	70.3^b^±6.0 (44)	53.8^b^±17.6 (3)	−0.44 (9.7)

1 LR-German Landrace, PiF1-(Pietrain × (German Large White × German Landrace), LW-German Large White.

2 Least-squares means (LSM) with different superscripts ^A,B; a,b^ differ significantly at p≤0.01 and 0.05 respectively.

3 Allele substitution effect in fractions of phenotypic standard deviation.

4 Phenotypic variance in percent explained by the SNP.

**Table 2:** Association of the three identified missense SNPs in porcine *NR3C1* with weight of the left adrenal gland (g) in three different commercial populations.

SNP	Population^1^ (n)	p-value	LSM ± SE^2^ (n)			a^3^ (% var^4^)
**c.39A>C**			AA	AC	CC	
	LR (673)	0.394	2.32±0.03 (658)	2.23±0.11 (15)		
	PiF1 (395)	0.889	2.30±0.03 (377)	2.29±0.08 (18)		
	LW (208)	0.002	2.30^A^±0.04 (193)	2.62^B^±0.10 (15)		0.82 (5.9)
**c.55G>C**			GG	GC	CC	
	PiF1 (395)	0.075	2.28±0.03 (250)	2.35±0.03 (145)		
**c.1829C>T**			CC	CT	TT	
	LR (673)	2.1E-39	2.42^A^±0.02 (563)	1.91^B^±0.04 (107)	1.76^B^±0.19 (3)	−1.23 (25.2)
	PiF1 (395)	4.3E-17	2.35^A^±0.02 (358)	1.94^B^±0.06 (31)	1.52^C^±0.12 (6)	−1.20 (22.4)
	LW (208)	1.6E-12	2.43^A^±0.04 (163)	1.91^B^±0.06 (43)	1.96^B^±0.23 (2)	−1.19 (28.2)

1 LR-German Landrace, PiF1-(Pietrain × (German Large White × German Landrace), LW-German Large White.

2 Least-squares means (LSM) with different superscripts ^A,B,C^ differ significantly at p≤0.01.

3 Allele substitution effect in fractions of phenotypic standard deviation.

4 Phenotypic variance in percent explained by the SNP.

The genotype-based analysis was complemented by haplotype-based association analysis. To examine whether the missense polymorphisms account for the previously found association of SNP c.*2122G>A with plasma cortisol level and adrenal weight, we also included genotype information of this SNP in the haplotype analysis (for more information see [8]). Based on these data, a total of five common haplotypes (frequency >1%) were inferred (Table S5). Each of the missense variants was located on a separate haplotype. We compared the four common haplotypes carrying allele A at the SNP c.*2122G>A, including the three haplotypes carrying the missense polymorphism, with the wild-type haplotype carrying the G allele, by means of the haplotype trend regression test. Estimated haplotype substitution effects are presented in Table 3. In accordance with the genotype-based analysis, haplotype A-G-T-A, carrying the derived T allele of SNP c.1829C>T, stood out as the only one showing consistent effects, significantly decreasing cortisol level and adrenal weight with similar magnitudes in all three populations. This finding suggests that SNP c.1829C>T is most likely underlying the previously found association of SNP c.*2122G>A with cortisol and adrenal weight [8].

**Table 3:** Haplotype effects of porcine *NR3C1* on plasma cortisol level (ng/ml) and adrenal weight (g) in three different commercial populations.

		Cortisol level		Adrenal weight	
Haplotype^1^	Population^2^	Substitution effect^3^	p-value	Substitution effect^3^	p-value
**A-G-C-A**	LR	−1.23±2.84	0.424	0.07±0.02	3.0E-4
	PiF1	0.01±2.60	0.998	−0.08±0.03	0.003
	LW	6.12±5.50	0.269	−0.03±0.06	0.583
**C-G-C-A**	LR	−0.49±6.73	0.942	−0.04±0.09	0.639
	PiF1	−9.45±6.29	0.134	0.06±0.08	0.421
	LW	5.50±8.74	0.531	0.21±0.09	0.021
**A-C-C-A**	PiF1	0.54±3.01	0.859	0.02±0.03	0.456
**A-G-T-A**	LR	−21.53±2.70	8.7E-15	−0.46±0.03	5.2E-35
	PiF1	−19.87±4.01	7.6E-7	−0.43±0.05	6.4E-18
	LW	−14.43±5.24	0.007	−0.44±0.06	1.4E-11

1 Haplotype c.39-c.55-c.1829-c.*2122.

2 LR-German Landrace (n = 673), PiF1-(Pietrain × (German Large White × German Landrace) (n = 391), LW-German Large White (n = 208).

3 Estimated substitution effects ± standard errors of indicated haplotypes compared to haplotype A-G-C-G.

Finally, we performed linkage disequilibrium (LD) and haplotype block analysis of the 3 Mb region between SNPs ALGA0106386 and ALGA0120126 on chromosome 2 using data from the GWAS on the 35 significant and 36 intervening SNPs, and SNP data from genotyping *NR3C1* in the LR population. The results are presented in Figure S2. The three segregating *NR3C1* SNPs, c.39A>C, c.1829C>T, and c.*2122G>A, formed a 102 kb haplotype block spanning the *NR3C1* gene, together with three GWAS SNPs, including the two most significant SNPs, ALGA0106239 and DRGA0017574. Among all analyzed SNPs, ALGA0106239 and DRGA0017574 showed the highest LD (r^2^ = 0.781) with SNP c.1829C>T.

Together, these results provide strong genetic evidence that SNP c.1829C>T is responsible for the association of *NR3C1* with plasma cortisol level and adrenal weight indicated in the present study by the GWAS and previously by the analysis of SNP c.*2122G>A [8].

### The p.Ala610Val substitution increases sensitivity of porcine GR to dexamethasone *in vitro*

**Figure 3:**
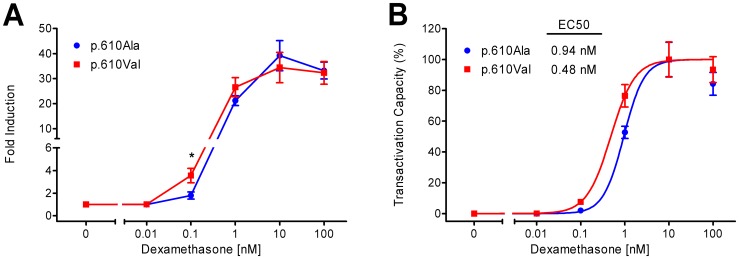
The p.Ala610Val substitution increases sensitivity of porcine GR to glucocorticoids *in vitro*. Transcriptional activity of the two GR-alpha variants was investigated in COS-7 cells cotransfected with the pGL4.36 reporter construct expressing firefly luciferase under the control of the glucocorticoid-inducible mouse mammary tumor virus promoter after induction with dexamethasone at the indicated concentrations. (A) Fold induction of normalized luciferase expression in dexamethasone- vs. vehicle-treated cells. Significant differences (p<0.05) are indicated by an asterisk; (B) Estimated dose-response curve and EC50. Data are means ± SEM of four separate experiments performed in triplicate.

As mentioned above, mutagenesis studies of GR indicated that the p.Ala610Val substitution might influence responsiveness of GR to glucocorticoids, which, in view of the key role of GR in feedback regulation of HPA axis activity, might explain phenotypic effects associated with SNP c.1829C>T. To examine the impact of the p.Ala610Val substitution on transcriptional activity of porcine GR, we compared the ability of the two allelic receptor variants to drive luciferase expression from the mouse mammary tumor virus promoter (MMTV) in COS-7 cells after stimulation with various dexamethasone concentrations (Figure 3). The transactivation assay revealed that the p.Ala610Val substitution enhances transcriptional activity of porcine GR in a dose-dependent manner (p = 0.007). The two GR variants have similar baseline transcriptional activity (Figure 3A). At 0.1 and 1 nM dexamethasone, respectively, the p.610Val variant induced 2-fold and 1.25-fold higher luciferase expression compared to the wild-type p.610Ala variant; however, only at 0.1 nM dexamethasone the difference reached significance (p = 0.012). At higher concentrations of dexamethasone, transcriptional activity reached a plateau that was similar for both GR variants. Analysis of the dose-response curves (Figure 3B) revealed that the half-maximal effective concentration (EC50) of dexamethasone was 2-fold lower for the p.610Val variant compared to the p.610Ala variant (0.48 nM and 0.94 nM, respectively; p = 0.026). These results demonstrate that the p.610Val variant of porcine GR has increased sensitivity to glucocorticoids compared to the p.610Ala variant and, thus, that SNP c.1829C>T is a gain-of-function mutation.

### Searching for functional coding and non-coding polymorphisms of *NR3C1* associated with SNP c.1829C>T

To further prove causality of SNP c.1829C>T, we investigated whether other polymorphisms in LD might affect function of *NR3C1* and, thus, potentially influence adrenal activity. We resequenced the region of *NR3C1* encoding the GR-alpha isoform in three additional individuals (one each PiF1, LR, and LW) homozygous for the A-G-T-A haplotype; apart from SNP c.1829C>T, no other coding polymorphisms (missense or synonymous) were found.

**Figure 4:**
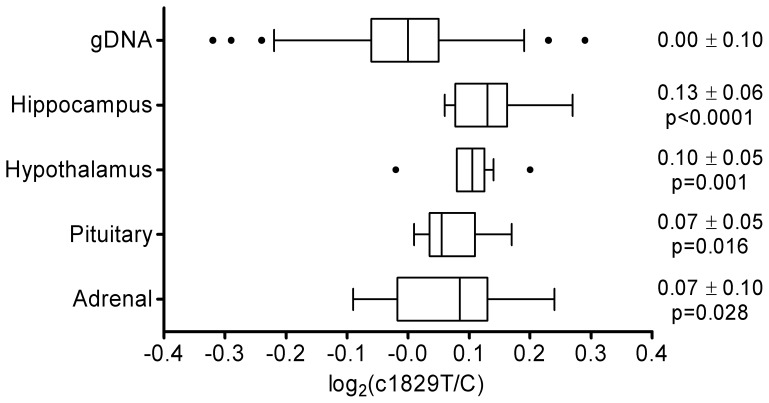
Allelic expression imbalance of porcine *NR3C1*. The box plots show the log_2_-transformed ratios of the T allele compared to the C allele of SNP c.1829C>T as measured in genomic DNA (gDNA) and in cDNA obtained from hippocampus, hypothalamus, pituitary, and adrenal tissues. The right side of the graph shows corresponding means ± standard deviations and p-values of the test for allelic expression imbalance.

To detect possible *cis*-acting effects of non-coding polymorphisms in LD with SNP c.1829C>T on expression of *NR3C1* in tissues related to the HPA axis, we analyzed its allelic expression imbalance (AEI) in hippocampus, hypothalamus, pituitary, and whole adrenal gland by pyrosequencing using ten samples per tissue (five samples for each diplotype A-G-T-A/A-G-C-G and A-G-T-A/A-G-C-A). In all four examined tissues, expression of the T allele was significantly higher compared to the C allele (Figure 4). However, the magnitude of the expression imbalance was very low, ranging between ∼1.05 [∼log_2_(AEI) = 0.07] and ∼1.10-fold [∼log_2_(AEI) = 0.13]. Further, allelic ratios in genomic DNA fluctuated tightly around one [i.e., log_2_(T/C)≈0.0], indicating that the porcine *NR3C1* locus is not affected by copy-number variation (Figure 4).

**Figure 5:**
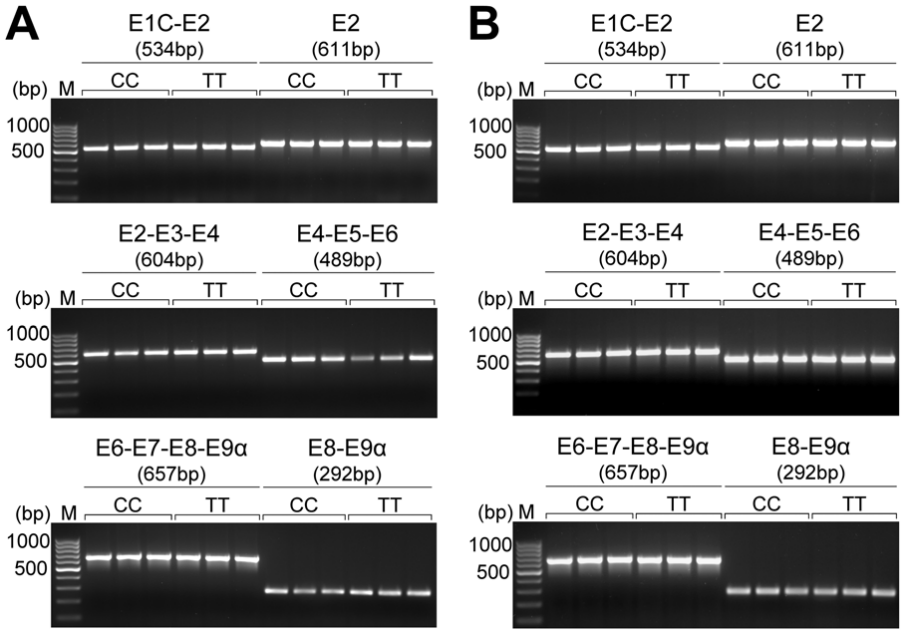
Splicing of porcine *NR3C1* shows no differences associated with SNP c.1829C>T. Splicing of the glucocorticoid receptor alpha isoform was examined using reverse-transcription PCR and cDNA from adrenal (A) and pituitary gland (B) of three individuals alternative homozygous for SNP c.1829C>T. Exons (denoted as E) of porcine *NR3C1* covered by the PCR amplicons are indicated above the corresponding PCR products.

Finally, to determine whether SNP c.1829C>T is in LD with a splicing polymorphism affecting structure of the GR-alpha isoform, we designed overlapping amplicons covering the cDNA sequence of *NR3C1* from a putative porcine homolog to human exon 1C (which shows widespread expression in humans [15]) and through exons 2–8 to exon 9-alpha (Figure 5). Using cDNA from adrenal gland and pituitary of three indviduals each homozygous for the carrier haplotype A-G-T-A and the wild-type haplotype A-G-C-G, the overlapping fragments were amplified, electrophoretically separated (Figure 5), and sequenced. We found no haplotype-specific differences in the splicing pattern, ruling out the existence of splicing polymorphisms in LD with SNP c.1829C>T.

Collectively, our data showed no considerable effects of non-coding polymorphisms in LD with SNP c.1829C>T on the function of porcine *NR3C1*, which leaves SNP c.1829C>T as the most likely causal variant.

## Discussion

Genetic studies of the inter-individual variation in HPA axis activity are comparatively rare; thus, knowledge of its molecular genetic background is limited in most species. Here, we contribute to the understanding of the genetics of this axis in the pig by identifying a polymorphism in *NR3C1* with a major effect on adrenal activity and by detecting several other genomic regions/QTL affecting plasma cortisol level and adrenal weight, most of which are novel.

Identification of a robustly replicating SNP-trait association is a crucial first step in identifying phenotypically causal genetic variants [16]. We show that a missense SNP c.1829C>T in *NR3C1* and the carrier haplotype are consistently and strongly associated with variation in plasma cortisol levels and adrenal weight in three populations with different breed origins. Importantly, not only direction, but also magnitudes, of the effects of SNP c.1829C>T were highly reproducible, implying either direct involvement of this SNP, or, at least, very tight linkage disequilibrium between the SNP and the causal variant.

Functional testing of candidate causal variants provides valuable clues to differentiate between phenotypically important and co-segregating, neutral genetic variant(s) [17]. Our experimental data demonstrated that SNP c.1829C>T is a functional mutation, increasing sensitivity of the porcine GR to glucocorticoids *in vitro* by about two-fold. Although the overall transactivation capacity of GR is not affected by the p.Ala610Val substitution, due to its increased sensitivity, the p.610Val variant becomes activated at lower glucocorticoid levels. This earlier activation could be expected to enhance feedback inhibition of the HPA axis activity and, consequently, lead to reduced secretion of ACTH, which is a major regulator of adrenal function and growth [18]. Inhibition of HPA axis activity is most likely persistent, as indicated by the reduction of adrenal weight associated with SNP c.1829C>T. A negative relationship between GR sensitivity and HPA axis activity was recently described by Zhang *et* *al.* [19] who observed markedly decreased basal and post-stress ACTH and corticosterone levels, reduced adrenal weight, and adrenocortical thinning in a knock-in mouse line carrying an artificial gain-of-function substitution, p.Met610Leu, in GR. Notably, the substitution engineered by Zhang *et* *al.* [20] is located in the GR-LBD next to the position corresponding with the p.Ala610Val substitution in the porcine GR-LBD (i.e., p.Ala611Val in murine GR). This substitution induces similar dose-dependent increases in transactivation activity of GR *in vitro* [20]. Hence, the p.Met610Leu knock-in mouse model provides substantial support for a causal role of SNP c.1829C>T.

However, in contrast to a knock-in mouse model, in an outbred species like the pig variation in a complex trait usually cannot be unambiguously attributed to a particular genetic variant, at least not before contribution of other variants in LD is considered. The only other detected missense polymorphisms of *NR3C1*, SNPs c.39A>C and c.55G>C, were located on different haplotypes and showed no or only inconsistent effects on adrenal activity. Consequently, supposing that other variants of *NR3C1* in LD with SNP c.1829C>T affect adrenal activity, these necessarily have to be functional non-coding polymorphisms. For their detection we followed an experimental approach and analyzed association of SNP c.1829C>T with allelic expression imbalance and splicing of *NR3C1* in tissues related to HPA axis. We found no evidence for splicing polymorphisms and only modest allelic expression imbalance of *NR3C1*, which argues against any significant contribution of polymorphisms in LD with SNP c.1829C>T to the variation in adrenal activity, especially in view of evidence from genome-wide association studies that non-coding variants tend to have weak phenotypic effects [17]. However, we cannot formally exclude existence of other contributing genetic variants acting in other temporo-spatial or functional contexts.

Taken together, our findings provide several mutually reinforcing lines of evidence supporting the conclusion that SNP c.1829C>T in porcine *NR3C1* is a gain-of-function mutation with a major effect on activity of the adrenal gland. This SNP is the first genetic variant in the pig for which a causal relationship to adrenal activity has been established and adds to a growing, but still limited, list of genetic variants known to be directly responsible for variation in complex traits (reviewed in [21]).

So far only two natural variants of GR, p.Asn363Ser and p.Asp401His (commonly designated N363S and D401H, respectively), that potentially increase its sensitivity to glucocorticoids have been described, both in humans; the majority of known natural GR variants are associated with glucocorticoid resistance [11]. The p.Asn363Ser and p.Asp401His variants of human GR are located in the N-terminal domain, both increase its transactivation capacity, and both are paradoxically associated with increased activity of the HPA axis [22], indicating that their mechanism of action is different from that of the porcine p.Ala610Val variant.

In the mouse, apart from the p.Met610Leu knock-in model, gene-targeting studies have been largely based on glucocorticoid receptor inactivation, which generally leads to an increase in the basal activity of the HPA axis [23]. Mice lacking GR (GR^NesCre^) in the nervous system display symptoms characteristic of Cushing syndrome, including increased glucocorticoid levels, reduced size, altered fat distribution, and reduced bone density [24]. In contrast, p.Met610Leu knock-in mice are phenotypically normal with respect to body composition or bone density [19]. In fact, phenotypic consequences of GR mutations are diverse, depending on which tissue or GR domain, i.e., signalling pathway, are affected. Because only a few natural or engineered GR variants with increased glucocorticoid sensitivity have been described, detailed phenotypic characterization of pigs carrying SNP c.1829C>T could offer new and–owing to the overwhelming physiological and genomic similarities between pigs and humans [25]–uniquely relevant insights into neurobiological and physiological consequences of genetically-based GR hypersensitivity. A particularly important aspect for future research is how the markedly decreased cortisol secretion affects stress susceptibility, adaptation potential, health, and welfare of carriers of SNP c.1829C>T. Because the SNP segregates in all major commercial pig breeds, this knowledge could prove useful for molecular breeding to improve their robustness.

## Materials and Methods

### Animals and phenotypes

#### Ethics statement

Animal care and tissue collection processes followed the guidelines of the German Law of Animal Protection, and the experimental protocol was approved by the Animal Care Committee of the Leibniz Institute for Farm Animal Biology (FBN, Dummerstorf, Germany).

Three commercial pig populations were used for sample and phenotype collection: a population of purebred German Landrace pigs (n = 834) and a population of German Large White (n = 274) pigs, both consisting only of barrows; and a Pietrain × (German Large White × German Landrace) cross consisting of barrows (n = 291) and females (n = 246). The average age at sampling was ∼170 days.

Sample collection was performed in the experimental slaughter facility of the FBN between 0930 and 1130 h. Tissues used for RNA preparation were quickly removed, dissected, frozen in liquid nitrogen and stored at −80°C. For dissection of brain areas of interest (hippocampus, hypothalamus, and amygdala) the stereotaxic atlas of the pig brain served as a reference [26]. For tissue sampling and weighing, left adrenal gland was taken.

A 50 ml sample of trunk blood was collected from each pig during the exsanguination in a plastic tube containing 1 ml of 0.5 M EDTA. After plasma preparation samples were stored at −80°C until use. Plasma cortisol levels (total) were determined in duplicate using commercially available enzyme-linked immunosorbent assay (DRG, Marburg, Germany) according to manufacturer's protocol. The intra- and inter-assay coefficients of variation were lower than 7.0% and 9.8%, respectively. An overview of analyzed phenotypes is given in Table S1. Before association analysis outliers with cortisol levels ≥200 ng/ml were removed. Remaining data were approximately normally distributed.

Samples of Pi and Du animals used for assessment of allele frequency and of Hardy-Weinberg equilibrium were also collected in the experimental slaughter facility of the FBN.

### RNA extraction and cDNA synthesis

Isolation of total RNA was performed using TRI reagent (Sigma, Taufkirchen, Germany). After DNaseI treatment (Roche, Mannheim, Germany), RNA was cleaned using the NucleoSpin RNA II Kit (Macherey-Nagel, Düren, Germany). Quantity and purity of RNA were determined using the NanoDrop ND-1000 spectrophotometer (NanoDrop, Peqlab, Germany), and integrity was checked on 1% denaturing agarose gels.

First-strand cDNA was synthesized using SuperScript III MMLV reverse transcriptase (Invitrogen, Karlsruhe, Germany) in a reaction containing 1.5 μg RNA, a mixture of 500 ng random hexamers (Promega, Mannheim, Germany) and 500 ng of oligo (dT)11VN primer, according to manufacturer's protocol.

### Detection of polymorphisms

Genotyping using the PorcineSNP60 BeadChip (Illumina Inc., San Diego, CA, USA) was performed in accordance with manufactureŕs protocol for the SNP Infinium HD assay (http://www.illumina.com). In brief, 200 ng of DNA were used for genome-wide amplification and subsequent fragmentation. DNA was hybridized to the 62,163 locus-specific 50mers covalently linked to the beads distributed on the surface of the microarray. Single-base extension of oligos on the BeadChip, using captured DNA as a template, was performed, incorporating detectable labels on the BeadChip. Signals of each wavelength were determined using an Illumina iScan that converted the images to intensity data. Intensity data for each SNP were normalized and assigned a cluster position and a resulting genotype with the GenomeStudio software (Illumina Inc.); a quality score was generated for each genotype. Samples with call rates <95% were removed. Markers were excluded if they had low minor-allele frequency (MAF) <5%. The average call rate for all samples was 99.8%±0.2.

The sequence of porcine *NR3C1* encoding GR-alpha was retrieved from NCBI from the sequence of BAC clone CH242-105G5 (Accession: CU928713). For resequencing, five overlapping fragments of ∼600 bp covering the target region were designed and amplified in a standard PCR mix containing genomic DNA or cDNA as template, 0.2 µM of each primer (Tables S6 and S7), 50 µM of each dNTP, and 0.5 U SupraTherm Taq Polymerase in 1× supplied PCR buffer containing 1.5 mM MgCl2 (Genecraft, Lüdinghausen, Germany). The temperature profile consisted of 40 cycles of denaturation at 95°C for 15°s, annealing at appropriate Ta for 60 s, and extension at 72°C for 60 s. PCR products were sequenced using Big Dye Terminator Cycle sequencing kit V3.1 (Applied Biosystems, Darmstadt, Germany) and analyzed on ABI 3130 automated sequencer. Sequence polymorphisms were detected using the Multiple SeqDoC online tool [27] (http://research.imb.uq.edu.au/seqdoc/multi.html).

For genotyping SNP c.39A>C and c.55G>C, PCR-RFLP assays were established. Polymorphic regions were amplified in a standard PCR-reaction as described above using primers GRf1 and GRr6, and 10 µl of amplification products were digested overnight using 2.5 U of *Mbo*II and 10 U *Bgl*I restriction enzymes, respectively, according to manufacturer's recommendations (Fermentas, St. Leon-Rot, Germany). Resulting RFLPs were analyzed on 2% ethidium bromide-stained agarose gels.

SNP c.1829C>T was genotyped using pyrosequencing. The polymorphic region was amplified using primers GRf7 (biotinylated) and GRr7 and as described above, except that DreamTaq Polymerase (Fermentas) was used. The sequencing reaction was performed using primer GRseq1 and the Pyro Gold Reagent Kit (Biotage, Uppsala, Sweden) in the PSQ 96MA Pyrosequencing instrument (Biotage, Uppsala, Sweden) according to manufacturer's instructions.

Genotyping of SNP c.*2122G>A was described previously [8]. Information on primers and amplicons designed to detect DNA polymorphisms are summarized in Tables S6 and S7.

### Expression constructs and transactivation assay

To construct GR expression plasmids, a 2.38 kb GR-alpha fragment was amplified from cDNA of two individuals homozygous for the c.1829C>T SNP using primers GR_C-Flag_fw and GR_wt_rev (Table S6) and the proofreading PrimeSTAR HS DNA Polymerase (MoBiTec, Göttingen, Germany). The *Not*I restriction sites, nested in the amplification primers, were used for insertion of the GR-alpha fragment into the pCMV-Tag 1 plasmid (Agilent Technologies, Waldbronn, Germany). The resulting expression plasmids, pCMV-GRA610 and pCMV-GRV610, were checked by sequencing for orientation and absence of mutations other than SNP c.1829C>T.

COS-7 cells were seeded in 96-well plates at 2.5×10^4^ cells/well in DMEM supplemented with 10% FBS. The next day, cells were co-transfected with 200 ng pCMV-GRA610 or pCMV-GRV610, 100 ng pGL4.36, and 2 ng pRL-SV40 using Lipofectamine 2000.

The pGL4.36 reporter (Promega) expresses firefly luciferase under the control of the glucocorticoid-inducible MMTV promoter. The constitutive expression of Renilla luciferase by the pRL-SV40 plasmid (Promega) was used to normalize for transfection efficiency.

Twenty-four hours after transfection, cells were treated with dexamethasone (Sigma) in different concentrations ranging from 0 to 100 nM, and, 24 h later, firefly and Renilla luciferase activities were measured under the use of the Dual-Glo Luciferase Assay System (Promega) in a DTX 880 Multimode Detector (Beckman Coulter, Krefeld, Germany). Four separate experiments were preformed in triplicate. The effect of the p.Ala610Val substitution on the normalized expression of the firefly luciferase in the transactivation assay was tested by ANOVA using the MIXED procedure of the SAS V9.2 software package (SAS Institute, Cary, USA). The model included fixed effects of GR variant, dexamethasone concentration, and their interaction. Estimated least-squares means (LSM) were compared using t-test, and p-values were adjusted by simulation. Dose-response curve and EC-50 were estimated using GraphPad Prism 5 (GraphPad Software Inc, San Diego, CA).

### Analysis of *NR3C1* transcription

To analyze allelic expression imbalance, the ratio of alleles of SNP c.1829C>T in cDNA and corresponding genomic DNA (gDNA) samples were measured using pyrosequencing. Pyrosequencing was performed as described above in two separate experiments, in duplicate. Ratios obtained from cDNA were normalized using ratios obtained from gDNA within each experiment. After log2 transformation, allelic expression imbalance was tested by comparing ratios obtained from cDNA with ratios obtained from gDNA using a two tailed t-test.

For the analysis of splicing of the GR-alpha isoform, six overlapping amplicons covering the whole open reading frame (ORF) were designed. The sequence corresponding to human exon 1C was retrieved from the sequence of BAC clone CH242-105G5. Reverse-transcription PCR was performed using standard conditions described in the previous paragraph, and products were visualized on 2% agarose gels. Identity of the amplicons was confirmed by direct sequencing. Information on primers and amplicons used for the analysis of the integrity of GR-alpha ORF are summarized in Tables S6 and S7.

### Population genetic and association analysis

Genome-wide and single-marker association analyses were performed using mixed linear models implemented in the JMP Genomics 5 and SAS V9.2 software packages (SAS Institute), respectively. The model included fixed effect of the SNP genotype and, to account for relatedness, the random effect of sire. In addition, for cortisol data the model included random effect of slaughter date and slaughter order as a covariate. For adrenal weight the model included body weight as a covariate. For both traits gender was included as a fixed effect for the analysis of the PiF1 population. LSM for *NR3C1* genotypes were compared by t-test, and p-values were adjusted by Tukey-Kramer correction. Allele substitution effects were estimated by linear regression on the number of copies of the derived allele. FDR (q-value, [28]) was computed using JMP Genomics 5.

Haplotype inference was performed using the expectation-maximization algorithm implemented in the HAPLOTYPE procedure of SAS V9.2. Haplotype trend regression was performed by including one variable for each haplotype indicating the number of copies of the haplotype in question in the models described above.

Hardy-Weinberg equilibrium was analyzed using the ALLELE procedure of SAS V9.2. Linkage disequilibrium between SNPs and haplotype blocks were determined using Haploview V4.2 [29]. Haplotype blocks were defined using the four gametes rule criteria.

## Supporting Information

Figure S1 **Alignment of the glucocorticoid receptor amino acid sequence among different vertebrates.** Two regions of the glucocorticoid receptor amino acid sequence are shown where DNA polymorphisms in the porcine *NR3C1* introduce amino acid substitutions. The position of the three variable residues found in pigs is indicated by arrows, and the homologous residues of other species by gray bars. Evolutionarily conserved residues are indicated by dots. Residues of helix 5 of the ligand binding domain are underlined.
(TIF)

Figure S2 **Linkage disequilibrium analysis of the QTL region on chromosome 2.** The values in the boxes show linkage disequilibrium between SNPs (r^2^) and the box color reflects the degree of linkage disequilibrium. A. Linkage disequilibrium analysis of 71 PorcineSNP60 BeadChip SNPs, including 35 genome-wide significant and 36 intervening SNPs along with three *NR3C1* SNPs, c.39A>C, c.1829C>T, and c.*2122G>A. B. Detail of the haplotype block harboring *NR3C1* SNPs c.39A>C, c.1829C>T, and c.*2122G>A.
(TIF)

Table S1 Descriptive statistics of the analyzed traits.
(DOC)

Table S2 SNPs with genome-wide significant evidence for association with plasma cortisol level.
(DOC)

Table S3 SNPs with genome-wide significant evidence for association with adrenal weight.
(DOC)

Table S4 Frequencies of the three identified missense SNPs in porcine *NR3C1* in commercial breeds.
(DOC)

Table S5 Frequencies of inferred haplotypes of porcine *NR3C1* in three different commercial populations.
(DOC)

Table S6 Primer information.
(DOC)

Table S7 Information on amplicons used for the analysis of splicing pattern, resequencing, and genotyping of *NR3C1*.
(DOC)
