# Supplementary figures and images for: A Substitution in the Ligand Binding Domain of the Porcine Glucocorticoid Receptor Affects Activity of the Adrenal Gland
Source: PLoS One. 2012 Sep 18;7(9):e45518. doi: 10.1371/journal.pone.0045518 (PMC3445511; doi:10.1371/journal.pone.0045518)

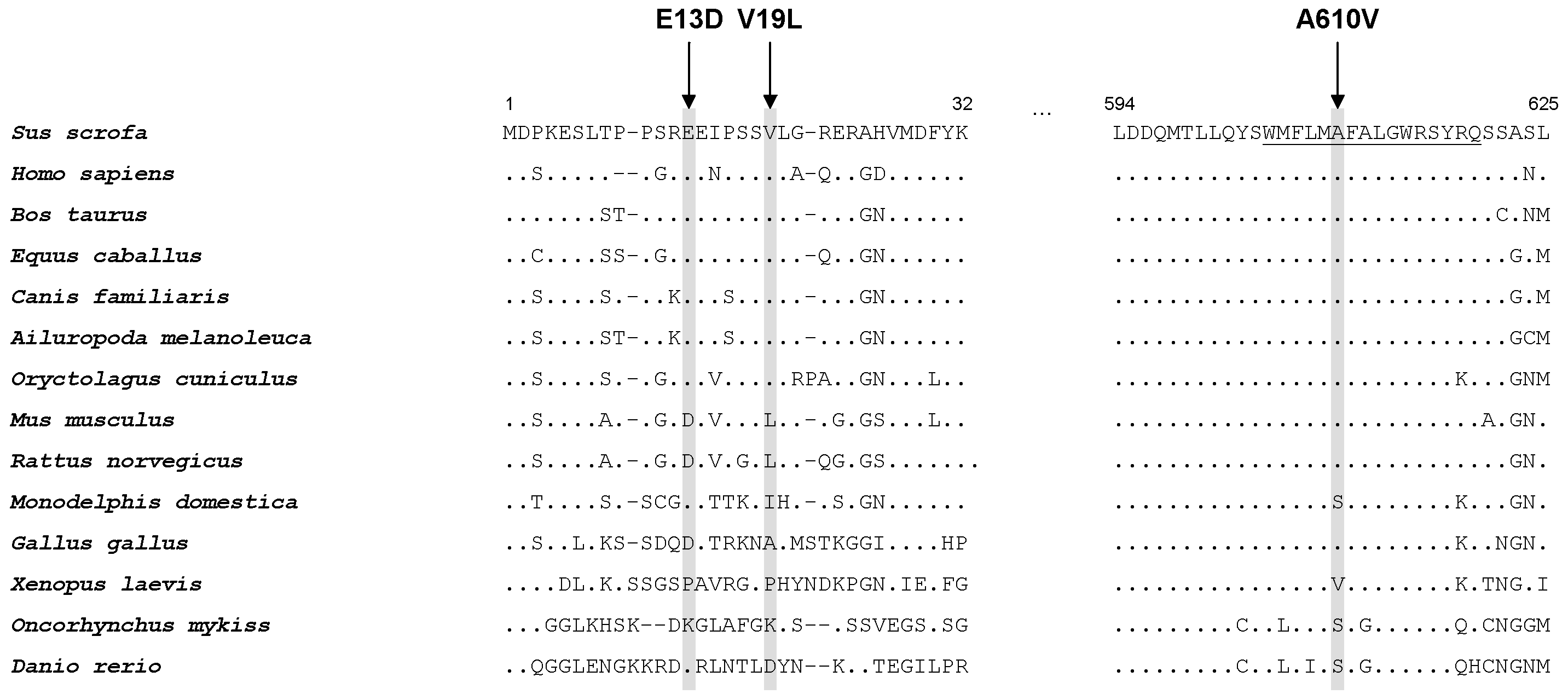

Supplement: Figure S1 — Alignment of the glucocorticoid receptor amino acid sequence among different vertebrates. Two regions of the glucocorticoid receptor amino acid sequence are shown where DNA polymorphisms in the porcine NR3C1 introduce amino acid substitutions. The position of the three variable residues found in pigs is indicated by arrows, and the homologous residues of other species by gray bars. Evolutionarily conserved residues are indicated by dots. Residues of helix 5 of the ligand binding domain are underlined. (TIF) [file pone.0045518.s001.tif]

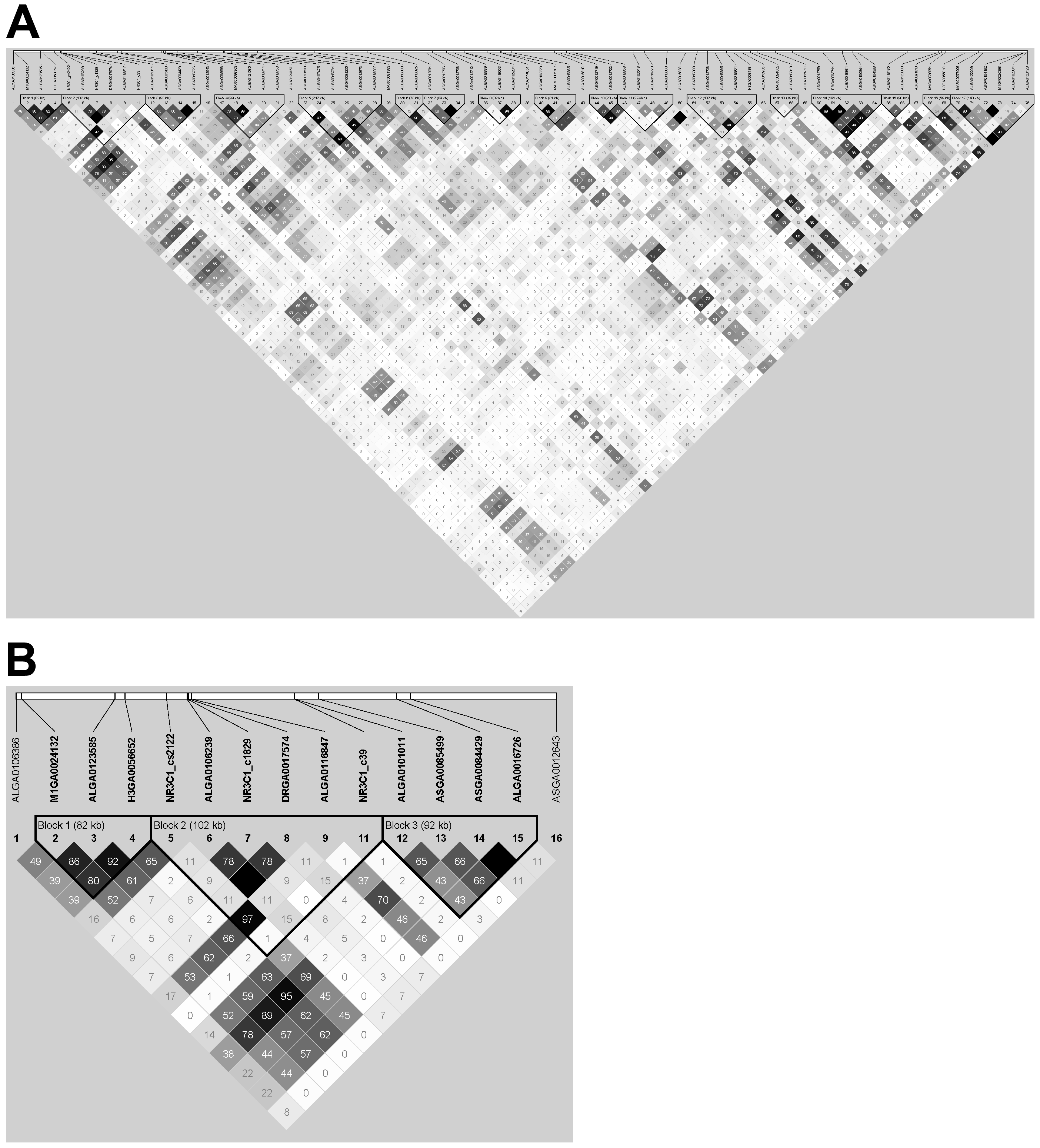

Supplement: Figure S2 — Linkage disequilibrium analysis of the QTL region on chromosome 2. The values in the boxes show linkage disequilibrium between SNPs (r2) and the box color reflects the degree of linkage disequilibrium. A. Linkage disequilibrium analysis of 71 PorcineSNP60 BeadChip SNPs, including 35 genome-wide significant and 36 intervening SNPs along with three NR3C1 SNPs, c.39A>C, c.1829C>T, and c.*2122G>A. B. Detail of the haplotype block harboring NR3C1 SNPs c.39A>C, c.1829C>T, and c.*2122G>A. (TIF) [file pone.0045518.s002.tif]
