# Supplementary material for: A Substitution in the Ligand Binding Domain of the Porcine Glucocorticoid Receptor Affects Activity of the Adrenal Gland
Source: PLoS One. 2012 Sep 18;7(9):e45518. doi: 10.1371/journal.pone.0045518 (PMC3445511; doi:10.1371/journal.pone.0045518)
Supplement: Table S1 — Descriptive statistics of the analyzed traits. (DOC) [file pone.0045518.s003.doc]

**Table S1. Descriptive statistics of the analyzed traits.**

| **Trait** | **Population**^1^ | **n** | **Mean** | **SD** |
| --- | --- | --- | --- | --- |
| **Cortisol (ng/ml)** | LR | 786 | 73.3 | 30.8 |
|  | PiF1 | 472 | 92.8 | 31.9 |
|  | LW | 233 | 84.5 | 34.7 |
|  |  |  |  |  |
| **Adrenal weight (g)** | LR | 673 | 2.36 | 0.40 |
|  | PiF1 | 395 | 2.31 | 0.34 |
|  | LW | 208 | 2.33 | 0.39 |

^1^ LR-German Landrace, PiF1-(Pietrain × (German Large White × German Landrace), LW-German Large White
