# Supplementary material for: A Substitution in the Ligand Binding Domain of the Porcine Glucocorticoid Receptor Affects Activity of the Adrenal Gland
Source: PLoS One. 2012 Sep 18;7(9):e45518. doi: 10.1371/journal.pone.0045518 (PMC3445511; doi:10.1371/journal.pone.0045518)
Supplement: Table S2 — SNPs with genome-wide significant evidence for association with plasma cortisol level. (DOC) [file pone.0045518.s004.doc]

**Table S2. SNPs with genome-wide significant evidence for association with plasma cortisol level.**

| **SNP_ID** | **Chromosome** | **Position**^1^ | **% Var**^2^ | **p-value** | **q-value**^3^ |
| --- | --- | --- | --- | --- | --- |
| **ALGA0004819** | 1 | 93194888 | 5.8 | 4.2E-05 | 0.046 |
| **ALGA0004829** | 1 | 93625456 | 5.8 | 4.2E-05 | 0.046 |
| **ALGA0004926** | 1 | 95685412 | 7.3 | 3.1E-06 | 0.007 |
| **ALGA0004933** | 1 | 96039450 | 5.9 | 4.0E-05 | 0.046 |
| **ALGA0106239** | 2 | 151278961 | 9.6 | 4.0E-08 | <0.001 |
| **DRGA0017574** | 2 | 151280257 | 9.6 | 4.0E-08 | <0.001 |
| **ALGA0016744** | 2 | 151619899 | 7.8 | 1.2E-06 | 0.004 |
| **ALGA0105204** | 2 | 152458829 | 5.8 | 4.6E-05 | 0.047 |
| **ALGA0114773** | 2 | 152984907 | 8.4 | 4.0E-07 | 0.002 |
| **ALGA0016868** | 2 | 153051045 | 6.3 | 1.7E-05 | 0.024 |
| **ALGA0016889** | 2 | 153138328 | 6.3 | 1.7E-05 | 0.024 |
| **ALGA0016913** | 2 | 153366943 | 6.0 | 3.3E-05 | 0.042 |
| **ALGA0123033** | 2 | 153843032 | 6.5 | 1.3E-05 | 0.020 |
| **M1GA0004043** | 3 | 11605650 | 6.8 | 8.3E-06 | 0.015 |
| **ASGA0099130** | 3 | 23755518 | 5.7 | 5.1E-05 | 0.050 |
| **ASGA0100009** | 3 | 26110294 | 6.6 | 1.1E-05 | 0.019 |
| **ALGA0022493** | 4 | 5342878 | 6.1 | 2.6E-05 | 0.034 |
| **H3GA0016255** | 5 | 40397918 | 8.3 | 4.4E-07 | 0.002 |
| **ASGA0025473** | 5 | 40533611 | 8.3 | 4.4E-07 | 0.002 |
| **ASGA0025477** | 5 | 40564833 | 8.3 | 4.4E-07 | 0.002 |
| **BGIS0001413** | 5 | 40586779 | 8.1 | 6.7E-07 | 0.002 |
| **ALGA0031782** | 5 | 40607095 | 8.1 | 6.7E-07 | 0.002 |
| **SIRI0001451** | 6 | 90575645 | 6.8 | 7.0E-06 | 0.014 |
| **H3GA0023063** | 7 | 117594837 | 9.7 | 3.9E-08 | <0.001 |
| **ASGA0036233** | 7 | 117653969 | 7.3 | 3.0E-06 | 0.007 |
| **ASGA0036244** | 7 | 117758074 | 7.5 | 2.2E-06 | 0.006 |
| **ASGA0036275** | 7 | 118017748 | 9.7 | 3.4E-08 | <0.001 |
| **MARC0011341** | 7 | 118230829 | 7.1 | 4.0E-06 | 0.008 |
| **ALGA0045079** | 7 | 121037142 | 4.9 | 3.7E-05 | 0.045 |
| **MARC0044680** | 7 | 122453069 | 5.5 | 1.3E-05 | 0.020 |
| **H3GA0024739** | 8 | 35145609 | 6.2 | 2.3E-05 | 0.032 |
| **ALGA0047484** | 8 | 35266477 | 6.2 | 2.3E-05 | 0.032 |
| **H3GA0024755** | 8 | 35394683 | 5.8 | 4.1E-05 | 0.046 |
| **ALGA0103800** | 8 | 35441596 | 4.8 | 4.8E-05 | 0.048 |
| **ALGA0049550** | 8 | 132026434 | 5.8 | 4.4E-05 | 0.046 |
| **ALGA0106042** | 16 | 18134454 | 6.8 | 7.2E-06 | 0.014 |
| **ALGA0089321** | 16 | 18600403 | 7.3 | 2.9E-06 | 0.007 |

^1^ Physical position on *Sus scrofa* genome build 10

^2^ Phenotypic variance in percent explained by the SNP

^3^ False discovery rate
