# Supplementary material for: A Substitution in the Ligand Binding Domain of the Porcine Glucocorticoid Receptor Affects Activity of the Adrenal Gland
Source: PLoS One. 2012 Sep 18;7(9):e45518. doi: 10.1371/journal.pone.0045518 (PMC3445511; doi:10.1371/journal.pone.0045518)
Supplement: Table S3 — SNPs with genome-wide significant evidence for association with adrenal weight. (DOC) [file pone.0045518.s005.doc]

**Table S3. SNPs with genome-wide significant evidence for association with adrenal weight.**

| **SNP_ID** | **Chromosome** | **Position**^1^ | **% Var**^2^ | **p-value** | **q-value**^3^ |
| --- | --- | --- | --- | --- | --- |
| **H3GA0053054** | 1 | 38011608 | 5.1 | 6.0E-05 | 0.039 |
| **ASGA0008705** | 2 | 5920823 | 8.6 | 4.9E-08 | <0.001 |
| **ALGA0123323** | 2 | 94712514 | 5.0 | 6.6E-05 | 0.041 |
| **M1GA0024750** | 2 | 148184334 | 10.2 | 1.8E-09 | <0.001 |
| **DRGA0003684** | 2 | 148905584 | 8.9 | 2.7E-08 | <0.001 |
| **ALGA0016635** | 2 | 149088667 | 10.0 | 2.6E-09 | <0.001 |
| **ALGA0106386** | 2 | 151142352 | 9.9 | 3.4E-09 | <0.001 |
| **M1GA0024132** | 2 | 151146729 | 8.3 | 8.1E-08 | <0.001 |
| **ALGA0123585** | 2 | 151221226 | 10.2 | 1.7E-09 | <0.001 |
| **H3GA0056652** | 2 | 151229225 | 8.7 | 3.7E-08 | <0.001 |
| **ALGA0106239** | 2 | 151278961 | 31.2 | 3.5E-31 | <0.001 |
| **DRGA0017574** | 2 | 151280257 | 31.2 | 3.5E-31 | <0.001 |
| **ALGA0101011** | 2 | 151364977 | 4.9 | 8.9E-05 | 0.049 |
| **ASGA0085499** | 2 | 151384081 | 8.3 | 8.6E-08 | <0.001 |
| **ASGA0089698** | 2 | 151580659 | 4.9 | 8.7E-05 | 0.049 |
| **ALGA0016744** | 2 | 151619899 | 17.2 | 4.4E-16 | <0.001 |
| **ALGA0124167** | 2 | 151755051 | 6.6 | 2.7E-06 | 0.003 |
| **ASGA0091508** | 2 | 151758763 | 6.5 | 3.6E-06 | 0.004 |
| **ALGA0016791** | 2 | 151800922 | 6.2 | 6.4E-06 | 0.006 |
| **ALGA0016777** | 2 | 151976644 | 6.0 | 9.3E-06 | 0.008 |
| **MARC0081393** | 2 | 152020938 | 7.5 | 4.4E-07 | 0.001 |
| **ALGA0016809** | 2 | 152101953 | 5.6 | 1.9E-05 | 0.015 |
| **ASGA0012691** | 2 | 152276319 | 5.6 | 1.9E-05 | 0.015 |
| **ALGA0119053** | 2 | 152458287 | 15.9 | 7.1E-15 | <0.001 |
| **ALGA0105204** | 2 | 152458829 | 16.3 | 3.4E-15 | <0.001 |
| **ASGA0103220** | 2 | 152564573 | 5.0 | 7.3E-05 | 0.043 |
| **ALGA0016865** | 2 | 152596156 | 5.0 | 7.3E-05 | 0.043 |
| **ALGA0016849** | 2 | 152722814 | 5.9 | 1.1E-05 | 0.010 |
| **ALGA0114773** | 2 | 152984907 | 13.8 | 8.7E-13 | <0.001 |
| **ALGA0016868** | 2 | 153051045 | 13.4 | 1.8E-12 | <0.001 |
| **ALGA0016889** | 2 | 153138328 | 13.4 | 1.8E-12 | <0.001 |
| **ALGA0016901** | 2 | 153223843 | 7.7 | 3.2E-07 | <0.001 |
| **ALGA0016910** | 2 | 153313828 | 8.9 | 2.8E-08 | <0.001 |
| **ALGA0016913** | 2 | 153366943 | 17.2 | 4.1E-16 | <0.001 |
| **ALGA0116165** | 2 | 153752480 | 10.0 | 2.7E-09 | <0.001 |
| **ALGA0123033** | 2 | 153843032 | 15.3 | 3.1E-14 | <0.001 |
| **M1GA0026651** | 2 | 153864690 | 10.5 | 9.1E-10 | <0.001 |
| **H3GA0055810** | 2 | 153924183 | 6.4 | 4.4E-06 | 0.004 |
| **MARC0077006** | 2 | 153959853 | 9.1 | 1.9E-08 | <0.001 |
| **ALGA0122209** | 2 | 153975634 | 10.6 | 6.9E-10 | <0.001 |
| **ALGA0120126** | 2 | 154103060 | 9.1 | 1.9E-08 | <0.001 |
| **ALGA0123873** | 2 | 157857916 | 5.1 | 5.3E-05 | 0.037 |
| **MARC0034221** | 4 | 1802828 | 6.8 | 1.8E-06 | 0.002 |
| **ASGA0017041** | 4 | 1930679 | 6.3 | 4.9E-06 | 0.005 |
| **ASGA0017123** | 4 | 2464749 | 6.9 | 1.4E-06 | 0.002 |
| **ASGA0017234** | 4 | 2898563 | 6.5 | 3.3E-06 | 0.003 |
| **MARC0020722** | 4 | 129161353 | 4.9 | 8.8E-05 | 0.049 |
| **H3GA0024980** | 8 | 77840237 | 5.6 | 2.0E-05 | 0.015 |
| **ALGA0051731** | 9 | 21027382 | 4.9 | 7.9E-05 | 0.045 |
| **ASGA0099485** | 9 | 29005805 | 5.3 | 3.8E-05 | 0.029 |
| **H3GA0026822** | 9 | 29066067 | 5.0 | 7.3E-05 | 0.043 |
| **ASGA0042217** | 9 | 29177203 | 5.0 | 7.3E-05 | 0.043 |
| **MARC0070230** | 9 | 29181083 | 5.0 | 6.0E-05 | 0.039 |
| **ALGA0052058** | 9 | 29188332 | 5.0 | 6.0E-05 | 0.039 |
| **MARC0021267** | 9 | 29209750 | 5.0 | 6.0E-05 | 0.039 |
| **M1GA0024594** | 9 | 63907705 | 5.0 | 6.5E-05 | 0.041 |
| **ALGA0055464** | 9 | 140427192 | 5.2 | 4.7E-05 | 0.034 |
| **ASGA0103519** | 14 | 78321926 | 6.2 | 5.7E-06 | 0.005 |
| **MARC0048112** | 16 | 7268650 | 5.7 | 1.7E-05 | 0.014 |
| **DRGA0015878** | 16 | 15332040 | 5.8 | 1.3E-05 | 0.011 |
| **ASGA0072888** | 16 | 33313251 | 5.2 | 4.5E-05 | 0.033 |
| **ALGA0090392** | 16 | 40235169 | 6.1 | 7.3E-06 | 0.007 |

^1^ Physical position on *Sus scrofa* genome build 10

^2^ Phenotypic variance in percent explained by the SNP

^3^ False discovery rate
