# Supplementary material for: A Substitution in the Ligand Binding Domain of the Porcine Glucocorticoid Receptor Affects Activity of the Adrenal Gland
Source: PLoS One. 2012 Sep 18;7(9):e45518. doi: 10.1371/journal.pone.0045518 (PMC3445511; doi:10.1371/journal.pone.0045518)
Supplement: Table S4 — Frequencies of the three identified missense SNPs in porcine NR3C1 in commercial breeds. (DOC) [file pone.0045518.s006.doc]

**Table S4. Frequencies of the three identified missense SNPs in porcine *NR3C1* in commercial breeds.**

| **SNP^1^** | **Position** | **Substitution^1^** | **LR^2^ (834)** | **LW^2^ (274)** | **Pi^2^ (28)** | **Du^2^ (21)** | **PiF1^2^ (537)** |
| --- | --- | --- | --- | --- | --- | --- | --- |
| **c.39A>C** | Exon 2 | p.Glu13Asp | 0.013 | 0.035 | 0.000 | 0.500 | 0.029 |
| **c.55G>C** | Exon 2 | p.Val19Leu | 0.000^3^ | 0.000^4^ | 0.339 | 0.000 | 0.186^5^ |
| **c.1829C>T** | Exon 6 | p.Ala610Val | 0.082 | 0.104 | 0.018 | 0.048 | 0.060^5^ |

^1^Designation according to the human genome variation society nomenclature; frequencies of alternative alleles are shown

^2^Breeds LR-German Landrace, LW-German Large White, Pi-Pietrain, Du-Duroc, PiF1-Pietrain × (German Large White × German Landrace) ; number of samples in parentheses

^3^Number of genotyped individuals n=127

^4^Number of genotyped individuals n=69

^5^Underline indicates that the genotype distribution significantly (p<0.05) departures from Hardy-Weinberg equilibrium
