# Supplementary material for: A Substitution in the Ligand Binding Domain of the Porcine Glucocorticoid Receptor Affects Activity of the Adrenal Gland
Source: PLoS One. 2012 Sep 18;7(9):e45518. doi: 10.1371/journal.pone.0045518 (PMC3445511; doi:10.1371/journal.pone.0045518)
Supplement: Table S5 — Frequencies of inferred haplotypes of porcine NR3C1 in three different commercial populations. (DOC) [file pone.0045518.s007.doc]

**Table S5. Frequencies of inferred haplotypes of porcine *NR3C1* in three different commercial populations.**

| **Haplotype** | **LR**^1^ | **PiF1**^1^ | **LW**^1^ |
| --- | --- | --- | --- |
| **c.39-c.55-c.1829-c.*2122** | n=1668^2^ | n=1074^2^ | n=548^2^ |
| **A-G-C-G** | 0.447 | 0.524 | 0.763 |
| **A-G-C-A** | 0.459 | 0.205 | 0.098 |
| **C-G-C-A** | 0.013 | 0.025 | 0.035 |
| **A-C-C-A** | 0.000^2^ | 0.184 | 0.000^2^ |
| **A-G-T-A** | 0.081 | 0.057 | 0.104 |

^1^ LR-German Landrace, PiF1-(Pietrain × (German Large White × German Landrace), LW-German Large White

^2^ number of chromosomes

^3^ Assuming frequency of allele C of SNP c.55G>C equals zero in this population
