# Supplementary material for: A Substitution in the Ligand Binding Domain of the Porcine Glucocorticoid Receptor Affects Activity of the Adrenal Gland
Source: PLoS One. 2012 Sep 18;7(9):e45518. doi: 10.1371/journal.pone.0045518 (PMC3445511; doi:10.1371/journal.pone.0045518)
Supplement: Table S6 — Primer information. (DOC) [file pone.0045518.s008.doc]

**Table S6. Primer information.**

| **Primer** | **Position** | **Sequence 5´ - 3´** |
| --- | --- | --- |
| GRe1Cf1 | exon 1C | TCCTGCTTTCACACGCTAAGTTG |
| GRf1 | intron 1 | GAACCAGAGTTACCTAAAGGGTTCG |
| GRf2 | exon 2 | GAAAGCATCGCAAACCTCAGTAG |
| GRf3 | exon 2 | ATCTTACCGAGCCCCAACAG |
| GRr1 | exon 2 | CTTCACATTCGGCTGCTCTG |
| GRe2r1 | exon 2 | GCAGCGGAGGCTGAACTCTTG |
| GRr2 | exon 2 | CCGTGAACAGAAATGGCAGAC |
| GRr6 | exon 2 | GGAGAGTGAAACTGCCTTGGAC |
| GRf4 | exon 3 | GACCACCTCCCAAACTCTGC |
| GRr3 | exon 3/4 | TGTGCTGTCCTTCCACTGCTC |
| GRe4f | exon 4 | TGTGCTGGAAGAAATGACTGTATC |
| GRe4r | exon 4 | TGATACAGTCATTTCTTCCAGCAC |
| GRf5 | exon 6 | CACCTGGATGACCAAATGACC |
| GRf7 | exon 6 | TTCAGGAACTTACACCTGGATGAC |
| GRr7 | exon 6 | GCTGGCACTTGATTGTCTGTATG |
| GRseq1 | exon 6 | CACCCCAGGGCGAAC |
| GRr4 | exon 6/7 | AGGGTAAAGCCATTCTCTGCTC |
| GRf6 | exon 8 | TCAGAACTGGCAACGCTTTTATCAAC |
| GRr5a | exon 9α | AACCGCTACAGGACAGATTGATAG |
| GR_C-Flag_fw |  | ATAATAGCGGCCGCCCACCATGGACCCCAAGGAATCGCTGAC |
| GR_wt_rev |  | ATAATAGCGGCCGCTCATCACTTTTGATGAAACAGAAGTTTTTTG |
