# Supplementary material for: A Substitution in the Ligand Binding Domain of the Porcine Glucocorticoid Receptor Affects Activity of the Adrenal Gland
Source: PLoS One. 2012 Sep 18;7(9):e45518. doi: 10.1371/journal.pone.0045518 (PMC3445511; doi:10.1371/journal.pone.0045518)
Supplement: Table S7 — Information on amplicons used for the analysis of splicing pattern, resequencing, and genotyping of NR3C1. (DOC) [file pone.0045518.s009.doc]

**Table S7. Information on amplicons used for the analysis of splicing pattern, resequencing, and genotyping of *NR3C1***

| **Analysis** | **Amplicon** | **Primer** | **Ta (ºC)** | **Amplicon size (bp)** |
| --- | --- | --- | --- | --- |
| **Splicing** |  |  |  |  |
|  | **Exon1C-Exon2** | GRe1Cf1 | 63 | 534 |
|  |  | GRe2r1 |  |  |
|  | **Exon2** | GRf2 | 60 | 611 |
|  |  | GRr2 |  |  |
|  | **Exon2-Exon4** | GRf3 | 60 | 604 |
|  |  | GRe4r |  |  |
|  | **Exon4-Exon6** | GRe4f | 60 | 489 |
|  |  | GRr7 |  |  |
|  | **Exon6-Exon9α** | GRf5 | 60 | 657 |
|  |  | GRr5a |  |  |
|  | **Exon8-Exon9α** | GRf6 | 60 | 292 |
|  |  | GRr5a |  |  |
| **Resequencing** |  |  |  |  |
|  | **Intron1-Exon2** | GRf1 | 60 | 631 |
|  |  | GRr1 |  |  |
|  | **Exon2** | GRf2 | 60 | 611 |
|  |  | GRr2 |  |  |
|  | **Exon2-Exon3/4** | GRf3 | 60 | 571 |
|  |  | GRr3 |  |  |
|  | **Exon3-Exon6/7** | GRf4 | 60 | 669 |
|  |  | GRr4 |  |  |
|  | **Exon6-Exon9α** | GRf5 | 60 | 657 |
|  |  | GRr5a |  |  |
| **Genotyping** |  |  |  |  |
| **c.39A>C** | **Exon1-Exon2** | GRf1 | 63 | 373 |
| **c.55G>C** |  | GRr6 |  |  |
| **c.1829C>T** | **Exon6** | GRf7 | 63 | 108 |
|  |  | GRr7 |  |  |
